# Unravelling Resistance: Integrating Metabolism, Epigenetics, Immunology, and Proteostasis in Strategies against Kirsten Rat Sarcoma Viral Oncogene Homolog-mutant Colorectal Cancer

**DOI:** 10.7150/ijbs.118831

**Published:** 2026-01-01

**Authors:** Jingyi Li, Na Song, Rui Ma, Xiujuan Qu

**Affiliations:** 1 Department of Medical Oncology, the First Hospital of China Medical University, Shenyang 110001, China.; 2 Key Laboratory of Anticancer Drugs and Biotherapy of Liaoning Province, the First Hospital of China Medical University, Shenyang 110001, China.; 3 Liaoning Province Clinical Research Center for Cancer, the First Hospital of China Medical University, Shenyang 110001, China.; 4 Clinical Cancer Treatment and Research Center of Shenyang, the First Hospital of China Medical University, Shenyang, China.

**Keywords:** *KRAS* mutation, KRAS inhibitor, colorectal cancer, drug resistance, tumor microenvironment

## Abstract

Colorectal cancer (CRC) has a high incidence and mortality rate globally, with approximately 40% of patients harboring Kirsten rat sarcoma viral oncogene homolog (*KRAS*) mutations. Patients with *KRAS* mutations exhibit poorer prognoses compared to those with* KRAS* wild-type tumors, often showing resistance to targeted therapies and chemotherapy. There is an acute need for innovative therapeutic strategies, particularly as KRAS^G12C^ inhibitors have been approved by the U.S. Food and Drug Administration (FDA) for second-line therapy in treating non-small cell lung cancer (NSCLC). Yet, their efficacy in CRC remains suboptimal due to the emergence of drug resistance. Current research on KRAS inhibitor resistance in CRC focuses mainly on single biological processes, leaving the complex interplay between cellular systems underexplored. This review synthesizes evidence across four key dimensions — metabolism, epigenetics, immunology, and proteostasis — to reveal multidimensional mechanisms of resistance. How these interconnected pathways work in synergy with signaling aberrations, genetic alterations, and translational modifications to reinforce resistance was investigated. The research also integrates recent advances in multi-targeted strategies, including combinations of KRAS inhibitors with metabolic or epigenetic modulators or immune checkpoint blockade. These findings provide potential strategies for overcoming resistance in *KRAS*-mutant CRC, addressing a critical gap in precision oncology.

## Introduction

Colorectal cancer (CRC) stands as the third most frequently diagnosed malignancy and accounts for approximately 10% of all new cancer cases globally 1. Although there are various regimens for CRC, such as surgical intervention, chemoradiotherapy, targeted therapy, and immunotherapy, the mortality rate remains high due to the challenges arising from postoperative recurrence and distant metastasis. Furthermore, a significant proportion (40%) of CRC patients exhibit mutations in the Kirsten rat sarcoma viral oncogene homolog (*KRAS*). It has been thought to be “undruggable” in the past, bringing great challenges to the treatment of CRC patients. In CRC, approximately 85% of *KRAS* mutations are concentrated at three key hotspots: codons 12, 13, and 61. The most frequently observed mutation is codon 12, particularly the G12D and G12V subtypes, which are the most prevalent. In contrast, G13D and G12C mutations follow in descending order of frequency 2, 3. The *KRAS* gene encodes a protein that is pivotal in regulating various essential cellular functions, including cell proliferation, differentiation, and apoptosis 4. *KRAS* mutations lead to sustained activation of *KRAS*-dependent pathways 5. Patients harboring *KRAS* mutations not only show a poorer prognosis and shorter overall survival (OS) but also demonstrate reduced sensitivity to standard treatment 6. Patients with *KRAS* mutations also show resistance to receptor tyrosine kinase (RTK) antagonists, including anti-epidermal growth factor receptor (EGFR) monoclonal antibodies. This resistance arises due to *KRAS* mutations locking the protein in a constitutively active guanosine triphosphate (GTP)-bound state. This renders it independent of upstream RTK signaling (*e.g*., EGFR) and thus unresponsive to EGFR inhibition 7-12. Moreover, inhibitors that target the downstream signaling cascade of *KRAS*, including the mitogen-activated protein kinase (MEK) inhibitor selumetinib, exhibit limited efficacy in CRC patients. Therefore, it is necessary to investigate treatment options for *KRAS*-mutant CRCs.

For years, *KRAS* mutation has been considered “undruggable” due to its unique intrinsic characteristics. Ongoing research involves mutant KRAS inhibitors, including sotorasib and adagrasib, which have received approval for the second-line therapy of advanced non-small cell lung cancer (NSCLC) 13, 14. Nevertheless, these inhibitors exerted suboptimal effects in CRC, with mechanisms of resistance appearing prevalent. Mechanistic studies have primarily focused on isolated pathways, such as mitogen-activated protein kinase (MAPK) reactivation or bypass signaling. However, the complex multidimensional crosstalk among cellular processes is generally overlooked. Specifically, emerging evidence indicates that metabolic reprogramming, epigenetic modifications, immune microenvironment remodeling, and proteostasis dysregulation all contribute to resistance. This critical gap limits our ability to design effective combinatorial strategies.

Therefore, to dissect the molecular basis of KRAS inhibitor resistance in CRC, this review innovatively integrates four critical dimensions and abnormal activation of the MAPK pathway of resistance. By studying their regulatory modalities, the research identifies novel therapeutic mechanisms of resistance and provides ideas for developing rational combination therapies to overcome resistance in *KRAS-*mutant CRC.

## The structure of the KRAS protein

As a constituent of the *RAS* gene family, the *KRAS* gene is categorized as a proto-oncogene, first identified in human lung cancer cells in 1982 15, 16. The* KRAS* gene encodes the KRAS protein, a small GTPase that is part of the broader RAS superfamily of proteins, which includes KRAS4A, KRAS4B, HRAS, and NRAS 17-19. The KRAS protein comprising 188 amino acid residues can be attached to the intracellular membrane through farnesylation, a modification facilitated by farnesyltransferase 20, 21. Characterized by a molecular weight of 21 kDa, the KRAS protein comprises six β strands and five α helices, with two main domains: a conserved G-domain and highly variable regions at the C-terminus. The G-terminal comprises a switch I exchange region, a switch II exchange region, and a P-ring. It is responsible for transducing downstream signals in combination with downstream signals and is then involved in regulating cell growth and development 22. The last four amino acid residues at the C-terminal are CAAX, which are the targets of post-translational modifications and are essential in binding to cell membranes 18. The KRAS protein transitions from an inactive form to an active form by binding to GTP and guanosine diphosphate (GDP), respectively. The activity of KRAS is primarily regulated by guanine nucleotide exchange factors (GEFs) and GTPase-activating proteins (GAPs). Under physiological conditions, the KRAS protein exists in an inactive state bound to GDP. When cells receive stimuli such as growth factors or other external signals, GEFs become activated. The activated GEFs bind to KRAS and promote the release of GDP, allowing the empty nucleotide binding site to bind GTP. Upon binding GTP, KRAS undergoes a conformational change, transitioning to an active state, which enables interactions with downstream effector molecules and signal transduction 23. In the GTP-bound state, KRAS enhances its intrinsic GTPase activity through interactions with GAPs. GAPs facilitate the hydrolysis of GTP, leading to the inactivation of KRAS and its return to the GDP-bound state. When the *KRAS* gene mutates, it disrupts GAP-mediated GTP hydrolysis, bringing KRAS into an activated state. The continuously activated KRAS will continue activating downstream signaling pathways through phosphorylation, stimulating cell migration and proliferation, and ultimately leading to tumorigenesis 24-28.

## Frequency and type of *KRAS* gene mutations

*KRAS* is the most frequently mutated gene within the *RAS* family, representing nearly 85% of all *RAS* gene mutations, with *NRAS* and *HRAS* following in prevalence. The *KRAS* mutation rate reaches the highest level in pancreatic ductal adenocarcinoma (PDAC), approximately 90%, and then in CRC patients with a mutation rate ranging from 30% to 50% 29. Furthermore, *KRAS* mutations are found in various other malignancies, including NSCLC, cholangiocarcinoma, and cervical cancer. In CRC, mutations at the 12^th^ codon of the *KRAS* gene are particularly associated with tumor invasion and metastasis 30, followed by codons 13, 61, 117, and 146 31. A retrospective analysis indicated that mutations in the G12C and G12V loci were related to poorer OS 32. Mutated KRAS can stimulate several downstream signaling pathways. These pathways contain the RAS-RAF-MEK-ERK cascade, the RAS-PI3K-AKT-mTOR cascade 33, 34, and the RAS-(RAL)-NF-kB pathway. All of which contribute to cellular proliferation and survival 35-38. Among the various *KRAS* mutants, the KRAS^G12C^ protein exhibits the highest intrinsic rate of hydrolysis of GTP. This leads to a greater proportion of KRAS remaining in an inactive conformation, thereby facilitating the binding of allele-specific inhibitors targeting the KRAS^G12C^ variant 39.

## KRAS inhibitors

*KRAS* has long been regarded as “undruggable” as it does not contain a classical pocket suitable for the occupation of small molecule inhibitor binding for allosteric inhibitors 17, 24, 40, 41. The binding affinity of the KRAS protein for guanosine GTP is exceptionally high, operating at the picomolar level. This makes it nearly impossible to develop small molecule inhibitors that compete with GTP binding 42. In addition, non-selective inhibition of both mutant and wild-type KRAS may result in the inhibition of normal signaling, further leading to potential toxicity, which limited the progress of this type of inhibitor. Research has shown that ablation of the *KRAS* gene in mouse models leads to myelomonocytic metaplasia, resulting in the inability of the mice to survive 43. Furthermore, the clinical trial of the pan-KRAS inhibitor BI-1701963 was ultimately discontinued by the sponsor due to the occurrence of fatal interstitial lung disease and limited efficacy, with the best response being stable disease 44. These studies highlight the importance of evaluating the potential toxic consequences of these non-selective inhibitors in clinical applications.

### KRAS^G12C^ inhibitors

Approximately 3% of CRC patients have KRAS^G12C^ mutations, and research into KRAS^G12C^ inhibitors has made certain preliminary progress. Recent research, however, has underpinned significant strides in the development of targeted therapies against mutant *KRAS*. Shokat *et al*. discovered a hidden pocket in the mutant KRAS^G12C^-GDP complex (switch-II pockets) adjacent to mutant cysteine 12 45 (mutation from glycine to cysteine). Hence, binding to mutant cysteine stabilizes KRAS in an inactive GDP-bound state. In contrast, this covalent approach does not suppress wild-type KRAS due to the absence of cysteine in the active site. With the discovery of the switch II pocket of the KRAS protein, research into KRAS inhibitors has made rapid progress. Among these, the development of KRAS^G12C^ inhibitors is the most advanced, with several drugs having entered the phase of clinical research. In contrast, inhibitors targeting other mutations, such as KRAS^G12V^ inhibitors, remain at a preclinical stage of research.

#### Sotorasib

Sotorasib is the first marketed KRAS^G12C^ inhibitor approved by the U.S. Food and Drug Administration (FDA) 46, 47. It can bind covalently and irreversibly to cysteine in the switch-II pocket region, preventing GEF-catalyzed nucleotide exchange and inhibiting the activation of downstream signaling cascades. Sotorasib demonstrated vigorous antitumor activity in somatic cells and patients 48. To assess the therapeutic effects of sotorasib, a Phase-I clinical trial involving 129 patients with advanced solid tumors that possess the* KRAS^G12C^* mutation was conducted. Among NSCLC patients, the objective response rate (ORR) was 32.2%, with 88.1% of patients achieving disease control. The median progression-free survival (PFS) was 6.3 months. In contrast, CRC patients showed a confirmed response rate of only 7.1%, although 73.8% experienced disease control, with a median PFS of 4.0 months 49. Furthermore, the CodeBreaK100 study, a Phase-II clinical trial, assessed the efficacy of sotorasib in a cohort of 62 patients diagnosed with *KRAS^G12C^*-mutant CRC, revealing an ORR of only 9.7% 50. These findings indicate that sotorasib demonstrates comparatively less efficacy in CRC patients than in NSCLC patients 51.

#### Adagrasib

Adagrasib is a selective and irreversible inhibitor specifically designed to target the *KRAS^G12C^* mutations by covalently binding to the mutant cysteine residue 52, 53. In the study of KRYSTAL-1, a total of 25 patients with* KRAS^G12C^*-mutant solid tumors were included, of whom 15 patients had NSCLC, eight patients (53.3%) achieved a partial response, two patients were diagnosed with CRC, and one achieved a partial response 54. In December 2022, the FDA granted authorization for use of adagrasib in the treatment of patients with locally advanced or metastatic NSCLC with *KRAS^G12C^* mutations who previously received standard systemic therapy 14. Given the limited number of CRC patients incorporated in the research of KRYSTAL-1, further investigation is warranted to determine definitively the efficacy of adagrasib in this patient population.

#### Other KRAS^G12C^ inhibitors

There are many unapproved KRAS^G12C^ inhibitors that have demonstrated antitumor activity in clinical studies and preclinical models. Divarasib is a covalent KRAS^G12C^ inhibitor. In a Phase-I clinical trial, divarasib showed better efficacy in NSCLC compared to CRC 55, 56. Among the NSCLC cohort, a definite ORR was found in 53.4% of patients, with a median PFS of 13.1 months. In contrast, a confirmed ORR of 29.1% was recorded among the CRC patients, and the median PFS was 5.6 months. JDQ443 disrupts downstream signaling by targeting the KRAS^G12C^ switch-II P loop and shows enhanced antitumor activity in xenograft models, particularly in combination with inhibitors targeting associated signaling pathways 57. JNJ-74699157 covalently binds to the KRAS^G12C^-GDP complex to inhibit downstream signaling, but its development is halted due to significant toxicity observed in clinical trials (with 60% of patients experiencing elevated creatine phosphokinase (CPK) levels) 58, 59. The selective KRAS^G12C^ inhibitor 143D shows significant antitumor activity and synergizes with SOS1/EGFR/MEK/ERK inhibitors in both *in vitro* and *in vivo* models 60. Recently, a research team designed a novel small molecule, inspired by natural products, remodels cyclophilin A (CYPA) to bind selectively the active state of KRAS^G12C^, disrupting oncogenic signaling and causing tumor regression in various cancer models 61. Additionally, AZD4747 targets mutant *KRAS^G12C^* and penetrates the central nervous system, making it suitable for use in the treatment of tumors with brain metastases 62. AZD4625 is a highly potent KRAS^G12C^ inhibitor with favorable pharmacokinetic properties 63. The compound 12VC1 can degrade active KRAS^G12C^ and KRAS^G12V^, showing potential for non-covalent therapies 64. Additionally, BI-2852 binds both active and inactive KRAS with nanomolar affinity, showing a novel mechanism of action 65. Furthermore, numerous registered clinical trials (both ongoing and planned) are exploring the potential of new KRAS inhibitors (Table 1).

Although significant progress has been made in the development of KRAS^G12C^ inhibitors, the differences in efficacy across various types of cancer (such as NSCLC and CRC) imply that different genetic backgrounds and tumor microenvironments play important roles in resistance. NSCLC patients respond significantly better to these inhibitors than CRC patients, indicating that specific biomarkers or signaling pathways may affect drug effectiveness. This warrants further investigation. In addition, clinical research data indicate that patients have a PFS of only 4 to 6 months, and clinical responses are not enduring. Further studies are needed to enhance drug efficacy. The results indicated that some inhibitors (such as JNJ-74699157) were discontinued due to their significant toxicity, highlighting the need to evaluate drug safety and tolerability during the development of new drugs. Future research must ensure the safety of treatment methods while improving their efficacy.

### KRAS^G12D^ inhibitors

*KRAS^G12D^* mutations were observed in approximately 10% to 17% of CRC individuals 5, 66. Nevertheless, in contrast to KRAS^G12C^, selective inhibition of KRAS^G12D^ presents greater challenges considering the necessity for inhibitors to exhibit sufficiently high affinity in their binding to KRAS^G12D^. There are currently no approved KRAS^G12D^ inhibitors. MRTX1133 is the first small molecule compound developed to bind KRAS^G12D^ in a non-covalent manner. Functionally, MRTX1133 reduced cell viability and inhibited crucial signaling molecules in* KRAS^G12D^*-mutant pancreatic and other cancer cell lines. Although MRTX1133 entered the clinical research stage, work thereon was halted at Phase I due to unstable pharmacokinetic data. HRS-4642, a novel KRAS^G12D^ inhibitor, demonstrated promising antitumor activity in a Phase-I trial, with potential synergy observed when combined with carfilzomib. Carfilzomib may act synergistically with HRS-4642 by downregulating Notch4 signaling. In addition, the combination of HRS-4642 and carfilzomib can reshape the TME, promoting the infiltration and activation of immune cells with anti-tumor properties 67. Weller's team designed a small molecule compound RMC-9805 targeting the mutated *KRAS^G12D^*. This compound creates a neomorphic protein-protein interface between CYPA, an abundant intracellular chaperone, and active RAS by binding to CYPA, thereby covalently modifying the D12 mutation located in the induced pocket at the interface. RMC-9805 reduced tumor growth in experimental models of lung, pancreatic, and CRC 68. These findings demonstrate a potential therapeutic strategy for *KRAS^G12D^*-mutant CRC 69.

### KRAS^G12V^ inhibitor

KRAS^G12V^ mutations were observed in approximately 10% of CRC individuals 70. Research into KRAS^G12V^ inhibitors remains in the preclinical stage. A research team developed a selective siRNA molecule targeting KRAS^G12V^, named EFTX-G12V. EFTX-G12V demonstrated significant anti-tumor effects in a mouse xenograft model harboring the KRAS^G12V^ mutations. Furthermore, treatment with EFTX-G12V resulted in a marked upregulation of granzyme B expression within the tumor immune microenvironment. In addition, the team found that the combination of EFTX-G12V and cetuximab improved both the depth and duration of response in *in-vivo* models of CRC 71.

### KRAS vaccine

Cancer vaccines play an essential role in preventing tumor cells from evading immune surveillance by enhancing the body's immune response 72. Vaccine therapy represents a promising novel approach for targeting* KRAS* mutations. Recently, Reilly's research team conducted a Phase-I clinical trial to assess the efficacy of a novel vaccine, ELI-0022P, in patients with colorectal and pancreatic cancers containing *KRAS* mutations. ELI-002 2P is a three-component lymph node-targeted vaccine comprising amphiphile (Amph)-modified G12D and G12R mKRAS long peptides as well as Amph-modified Toll-like receptor 9 agonistic CpG-7909 DNA. Vaccination can robustly re-program the immune microenvironment to develop high-magnitude, functional T cell responses. The regimen was well tolerated, with 84% of patients showing tumor biomarker responses. The median recurrence-free survival was 16.33 months 72, 73. Furthermore, a seven-peptide product, ELI-002 7P has shown antitumor activity in patients with *KRAS*-mutant CRC and PDAC, inducing CD4 and CD8 responses in 63.6% of patients 74.

### KRAS degradation

In addition to covalently suppressing mutant KRAS proteins, the degradation of the oncogene KRAS using the proteolysis-targeting chimeras (PROTAC) has emerged as an alternative approach to *KRAS*-mutant cancers 75. PROTACs are bifunctional molecules including a ligand that recruits E3 ubiquitin ligases, a ligand that binds to the target protein, and a linker connecting these two components. This design directs the targeted protein to the E3 ligase for ubiquitination, followed by subsequent degradation by the proteasome 76, 77. Yang's research team designed a PROTAC known as YF135, which can induce quick and persistent degradation of endogenous KRAS^G12C^ and suppress pERK in *KRAS*-mutant CRC cell lines 78. Additionally, Zhou's team designed a compound, 8o, which can induce rapid degradation of KRAS^G12D^ and inhibit the proliferation of multiple* KRAS^G12D^*-mutant cancer cells including PDAC, NSCLC, and gastric cancer cells 79. Alessio's team discovered a novel pan-RAS inhibitor, ACBI3, which can degrade 13 of the 17 most common oncogenic *KRAS* alleles. Compared with traditional covalent inhibitors, ACBI3 exerts a more durable inhibitory effect on downstream signaling pathways 80.

## Mechanisms of resistance and reversal strategies of KRAS inhibitors in patients with CRC

The *KRAS*-mutant protein is considered to be constitutively active. *KRAS*-mutant tumors are independent of RTK activation. However, drug resistance to KRAS inhibitors in CRC involves multiple complex mechanisms, which can be classified into genetic alterations, reactivation of signaling pathways, and proteostasis reprogramming, as detailed below.

### Feedback activation of upstream signaling pathways

Reactivation of signaling pathways, particularly through EGFR, is a key resistance mechanism in *KRAS*-mutant cancers. EGFR/ERBB1 is the most characteristic RAS signaling activator, which acts by binding to the growth factor receptor binding protein 2 (GRB2) 81, 82. The GRB2 protein recruits and activates SOS1 and SHP2, activating the RAS signaling pathway 83-85. Co-targeting of KRAS and EGFR monoclonal antibodies was found to downregulate the level of RAS-GTP in both mutant and wild-type types 86.

Studies found that EGFR signaling significantly contributes to resistance against KRAS^G12C^ and KRAS^G12D^ inhibitors, suggesting that co-targeting KRAS and EGFR monoclonal antibodies may provide a new therapeutic approach to overcome resistance 87, 88. A study found that MRTX1133 exhibited synergistic anti-tumor activity when combined with pan-HER inhibitors in *KRAS*^G12D^-mutant CRC cells 89.

### Secondary mutations and gene alterations in resistance

Whole-genome sequencing of patients treated with KRAS inhibitors has uncovered various secondary mutations and fusions contributing to drug resistance. Notable alterations include: *MET* amplification, mutations in *RET*, *BRAF*, *MAP2K1*, and *NRAS*, as well as oncogenic fusions involving *ALK*, *FGFR3*, *BRAF*, *RAF1*, *RET*, and loss-of-function mutations in *PTEN*, and* NF1*. It is noteworthy that secondary *KRAS* mutations are also present in patients with drug resistance, such as *KRAS*^G12D^, *KRAS*^G12V^,* KRAS*^G13D^, among others. The acquired activation of the RTK-RAS-MAPK-PI3K cascade network and the emergence of specific fusion genes further demonstrate the complexity of mechanisms governing resistance faced by *KRAS*-mutant CRC tumors 90. These fusion genes encompass *CCDC6-RET*,* EML4-ALK*, *FGFR3-TACC3*,* AKAP-BRAF*,* NRF1-BRAF*,* RAF1-CCDC176*, and* RAF1-TRAK1*.

Genetic alterations are among the most thoroughly studied mechanisms of resistance in clinical practice, thus serving as actionable targets for personalized therapy. For instance, patients with *MET* amplification or *RET* fusions are likely to benefit from a combination therapy with KRAS and MET/RET inhibitors.

### Roles of the cell cycle and apoptosis pathways

**Figure 1:**
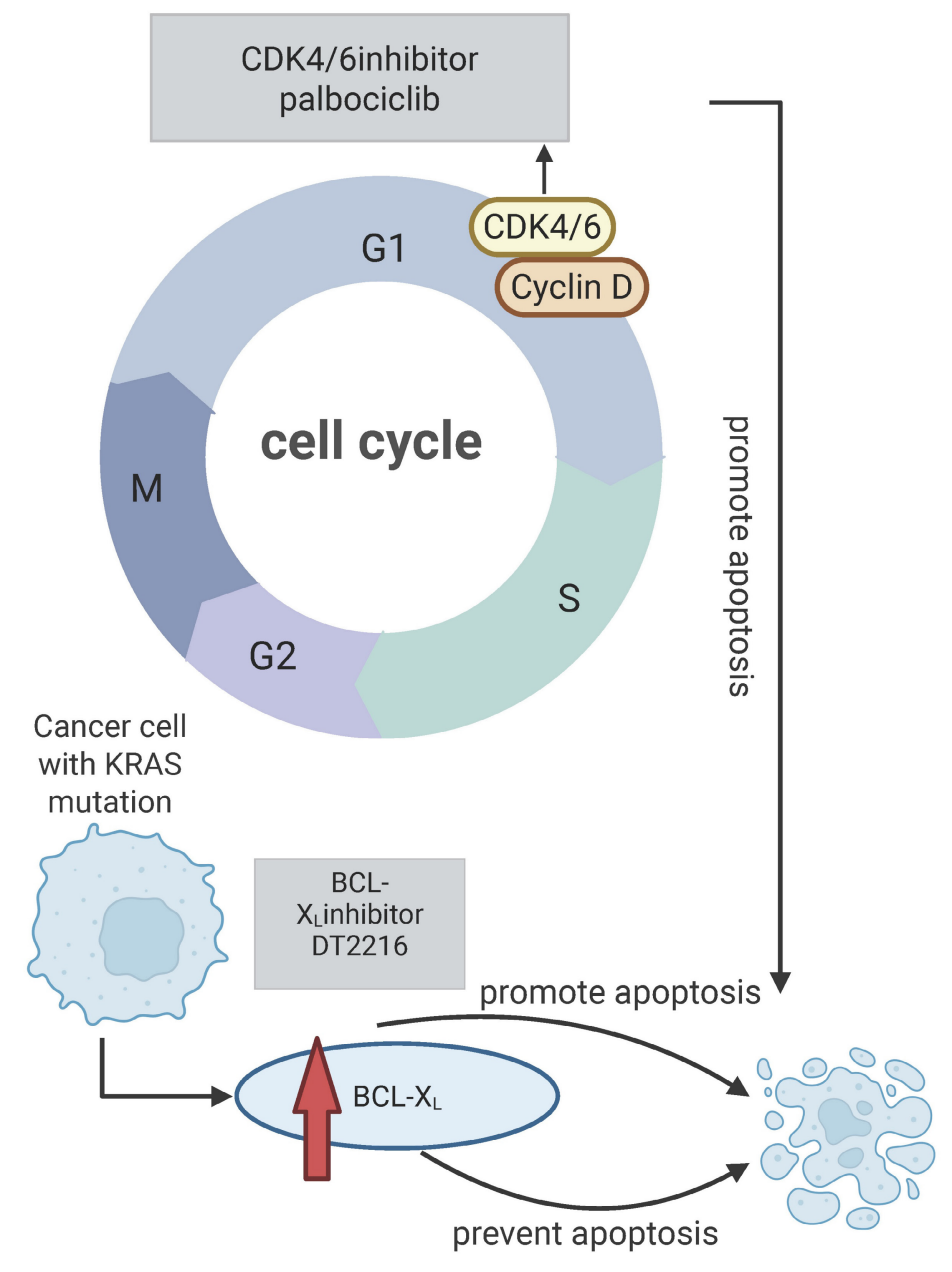
Effects of *KRAS* mutations on cell cycle and apoptosis. *KRAS* mutations can cause the level of BCL-XL protein, and then prevent apoptosis, targeting BCL-XL can promote apoptosis and reverse drug resistance, and CDK4/6 inhibitors can promote apoptosis by inhibiting cell cycle progression and have synergistic anti-tumor effects with* KRAS* inhibitors. Created in BioRender. Li, J. (2025) https://BioRender.com/vzgd3pj.

Resistance to KRAS^G12C^ inhibitors may involve alterations in key regulators of the cell cycle and apoptosis pathways. CDK4/6 promotes the cell cycle progression by phosphorylating the retinoblastoma gene (RB). A synthetic lethal relationship between CDK4 and KRAS has been identified in NSCLC models 91, 92. Additionally, high expression of the anti-apoptotic protein BCL-XL has been linked to resistance against KRAS^G12C^ inhibitors in *KRAS*-mutant tumors 93. Traditional BCL-XL targeting has resulted in severe thrombocytopenia, but the PROTAC DT2216 can selectively degrade BCL-XL without this side effect 94-96. Sotorasib facilitates the interaction between BCL-XL and BIM. Consequently, when DT2216 degrades BCL-XL, it can enhance the apoptosis of tumor cells alongside sotorasib treatment. Thus, simultaneously targeting BCL-XL and KRAS^G12C^, or co-targeting CDK4 and BCL-XL, may provide a novel strategy to overcome resistance and improve treatment outcomes for *KRAS*-mutant patients. Figure 1 illustrates the impact of *KRAS* mutations on apoptosis and the synergistic antitumor activity achieved by combining KRAS inhibitors with cell cycle or anti-apoptotic inhibitors 97.

### Proteostasis reprogramming

Protein homeostasis plays a crucial role in cell growth and development. Inactivation of the oncogene* KRAS* downregulates the heat-shock response and the IRE1α branch of the unfolded protein response (UPR), incurring severe disruption of protein homeostasis. However, cancer cells can employ mechanisms to restore protein homeostasis. Targeting these pathways may enhance cell death and reduce drug resistance. Wang's research team found that the HSP90 inhibitor AUY922 suppresses the growth of* KRAS*-mutant CRC xenografts by inducing apoptosis associated with endoplasmic reticulum (ER) stress. Mechanistically, AUY922-induced ER stress upregulates BIM transcription in *KRAS*-mutant CRC. BIM deficiency blocks the inhibitory effect of AUY922 on the growth of *KRAS*-mutant CRC xenografts. Furthermore, inhibiting GRP78 or XBP-1 - two key pro-survival factors in the ER stress response - enhances the cytotoxic effects of AUY922 98. Another research team found that, in *KRAS*-mutant CRC cell lines, gene ablation of ERN1 enhances cellular sensitivity to MEK inhibition. ERN1 is linked to the JUN pathway through its binding factor TRAF2, which mediates a signaling cascade that activates JUN N-terminal kinase (JNK), promoting cell proliferation. JNK kinase inhibitors exhibit synergistic antitumor activity in combination with MEK inhibitors 99. Tan's research team found that the expression levels of Nrf2 and GLS1 proteins were significantly elevated in CRC cell lines with acquired resistance to adagrasib. Subsequently, they extracted a natural cyclic peptide, RA-V (a natural cyclopeptide isolated from the roots of *Rubia yunnanensis*). RA-V can restore the sensitivity of resistant CRC cells to sotorasib. The specific mechanism of action involves RA-V inhibiting Nrf2 protein through ubiquitin-proteasome-dependent degradation, causing the induction of oxidative stress, ER stress, and DNA damage in CRC cell lines. Therefore, RA-V may serve as a novel therapy with which to improve the efficacy of adagrasib 100.

### Other common mechanisms of drug resistance

**Figure 2:**
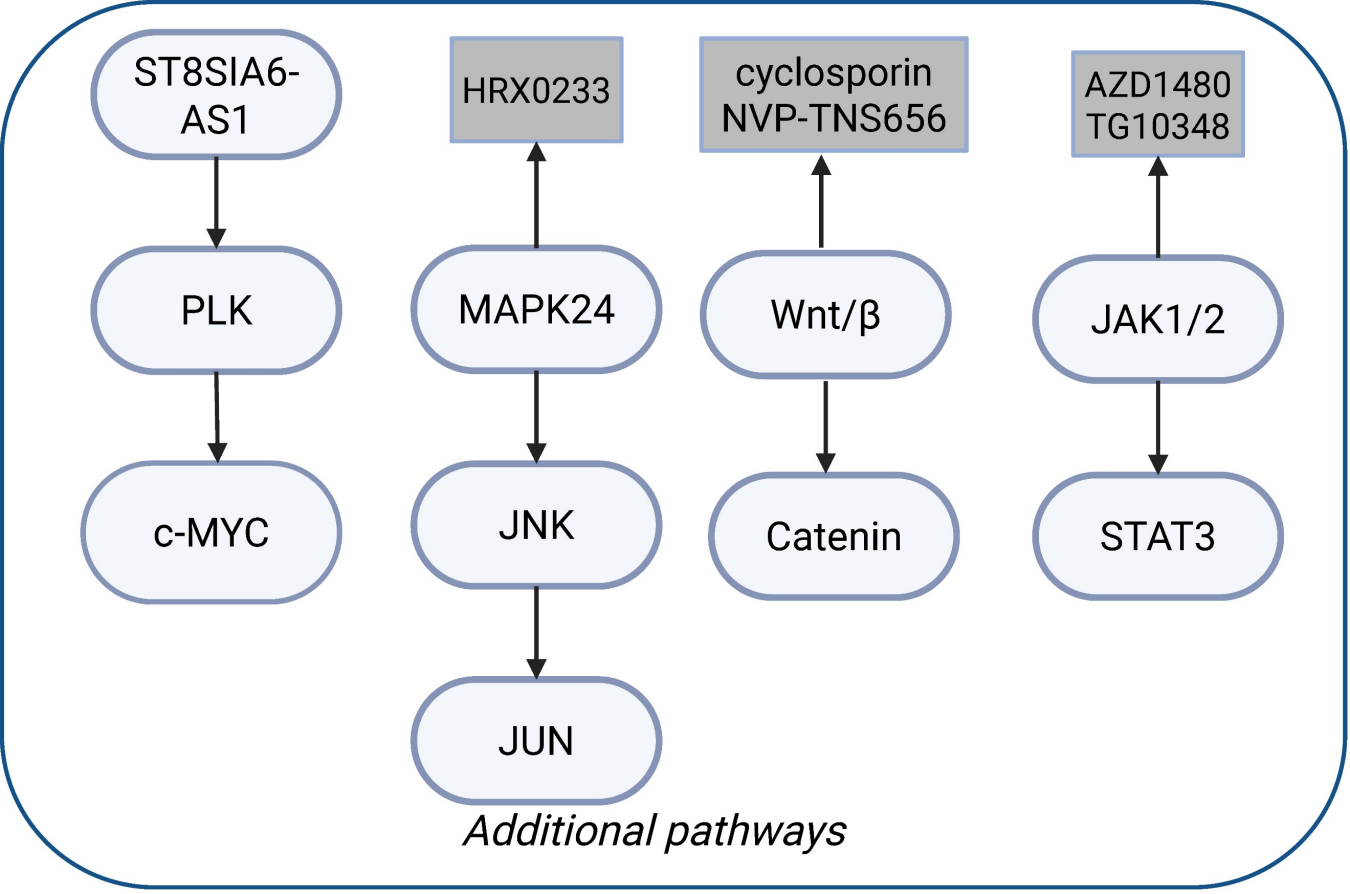
Common signaling pathways associated with drug resistance. Activation of the ST8SIA6-AS-PLK-c-MYC, MAPK24-JNK-JUN, Wnt/β-Catenin, and JAK1/2-STAT3 pathways is associated with drug resistance in *KRAS* mutated tumors, and targeting these pathways can reverse drug resistance. Created in BioRender. Li, J. (2025) https://BioRender.com/vzgd3pj.

In addition to the aforementioned mechanisms of resistance, the activation of other pathways is also associated with tumor drug resistance. Figure 2 shows that the activation of several common signaling pathways can lead to resistance to KRAS inhibitors. Polo-like Kinase (PLK1) and c-Myc are overexpressed in resistant cell lines, with PLK1 phosphorylating c-Myc. This activated c-Myc enhances the expression of PLK1 through E-box binding, creating a positive feedback loop that accelerates cell mitosis and tumor proliferation. ST8SIA6-AS1, a long non-coding RNA upregulated in drug-resistant cells, interacts with PLK1 and Aurora A, promoting PLK1 phosphorylation and reinforcing this feedback loop. Targeting ST8SIA6-AS1 can counteract resistance to KRAS^G12C^ inhibitors by disrupting the mutual activation of PLK1 and c-Myc signaling 101.

Another study highlighted the role of MAP2K4-JNK-JUN pathway in CRC resistance to KRAS^G12C^ inhibitors (Figure 2). MAP2K4 induces RTK-dependent rebound activation of KRAS signaling when MAPK inhibitors are present. JUN activation stimulates several RTKs, notably ERBB2 and ERBB3, reactivating the MAPK pathway and contributing to drug resistance. Combining sotorasib with the MAP2K4 inhibitor HRX-0233 can impede this feedback and enhance the antitumor activity 102, 103.

Johnson *et al*. found that the YAP1/TAZ pathway promotes resistance in *KRAS*-mutant patients 104. YAP1/TAZ can regulate transcription through TEAD, stimulating the ERK-independent gene expression that promotes cell cycle progression and activates PI3K-AKT-mTOR signaling, allowing cells to evade apoptosis 105. These findings emphasize the complex mechanisms of drug resistance in *KRAS*-mutant CRC, potentially providing new therapeutic strategies for clinicians. By disrupting these feedback loops and signaling pathways, the efficacy of KRAS^G12C^ inhibitors may be enhanced.

The above studies indicate that the resistance mechanisms in *KRAS*-mutant CRC are dynamic and multifaceted processes, involving feedback activation, acquired genetic changes, and reprogramming of core cellular processes such as protein homeostasis and apoptosis. This suggests that merely targeting KRAS is insufficient. These findings facilitate a shift in treatment paradigms from monotherapy to mechanism-based combination therapies. Future directions should focus on proactively targeting these escape pathways. Additionally, identifying reliable biomarkers to pinpoint the dominant resistance pathways in patients is crucial.

## Reversal of KRAS inhibitor resistance by targeting downstream signaling pathways

**Figure 3:**
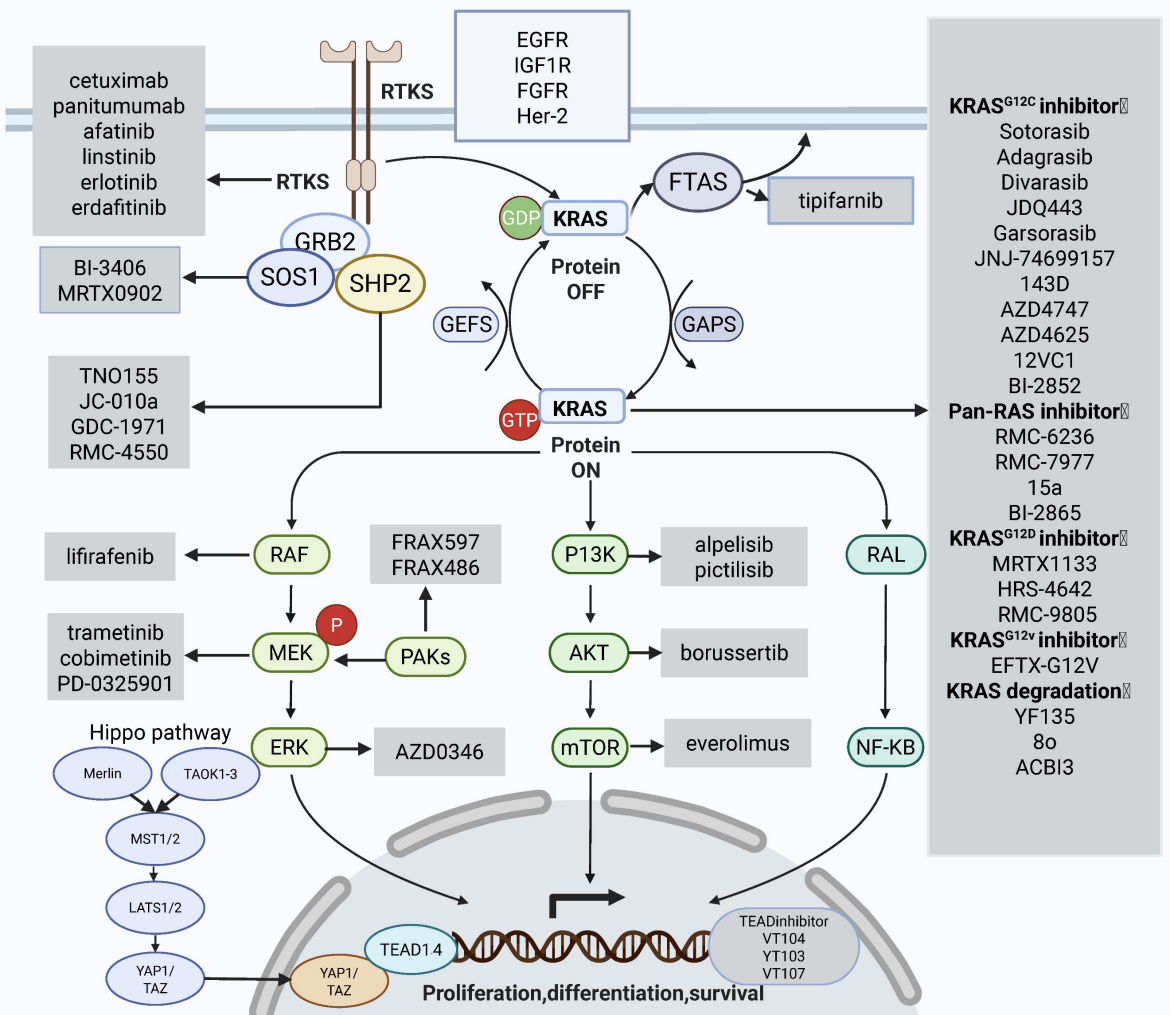
KRAS signaling pathways and combination therapies that can alleviate resistance. The main mechanisms that can reverse drug resistance are: targeting the downstream RAF-MEK-ERK signaling pathway; targeting the PI3K-AKT signaling pathway; targeting YAP/TAZ and its transcription factor TEAD in the Hippo pathway, targeting upstream SHP2, SOS1, FTAS, targeting upstream continuously activated receptor tyrosine kinases. The gray box areas show the main targeted drugs for each signaling pathway. Created in BioRender. Li, J. (2025) https://BioRender.com/vzgd3pj.

Due to the limited efficacy of KRAS inhibitors as monotherapy, resistance often develops rapidly; therefore, the development of combination therapies is progressing swiftly. As shown in Figure 3, common combination therapies that can alleviate resistance are summarized; these are based on the RAS signaling pathway and focus on its downstream and upstream components.

### Targeting the MAPK signaling pathway

A prominent mechanism underpinning drug resistance is the reactivation of the RAS-MAPK pathway 106. The MEK is an important component of the RAF/MEK/ERK cascade downstream of KRAS 107. For a long time, MEK inhibitors have failed to achieve any significant efficacy in CRC patients 108. This phenomenon might stem from feedback phosphorylation of MEK by C-RAF and reactivation of MAPK signaling. However, MEK inhibitors lay a basis for combination therapy, showing synergistic effects when paired with RAF inhibitors 109. Xi *et al*. reported that the RAF dimer inhibitor lifirafenib showed strong synergy with MEK inhibitors in suppressing the proliferation of *KRAS*-mutant CRC cell lines 110. Other research found that the combination of type II RAF inhibitors with MEK inhibitors could overcome acquired resistance in *KRAS*-mutant cancers. Additionally, resistance of MEK inhibitors in CRC is found to be correlated with ERK-dependent activation of the RTK-SOS-WTRAS-PI3K signaling pathway 111-113. MEK inhibitors, when combined with ERK inhibitors, exert a synergistic effect in* KRAS*-mutant tumors and significantly reverse the resistance caused by MEK inhibitors alone 114. Research has identified that in CRC cell lines exhibiting resistance to MEK inhibitors, the GRB7-mediated signaling pathway is prominently enriched. PLK1 functions as a major interacting kinase with GRB7; thus, inhibition of PLK1 can suppress the downstream signaling associated with RTKs, thereby reversing drug resistance 115.

### Targeting the PI3K-AKT-mTOR signaling pathway

The PI3K/AKT signaling cascade was confirmed to be activated in sotorasib-resistant cell lines, with sotorasib and PI3K inhibitor alpelisib showing synergistic cytotoxicity 116. Belli *et al*. found that mTOR inhibitor everolimus and PAK inhibitor exhibit synergistic antitumor activity in *KRAS*-mutant CRC 117. Targeting the MAPK signaling pathway and the PI3K-AKT-mTOR pathway will be efficacious 118. However, these combination strategies may also cause increased toxicity profiles. A Phase-I clinical study showed that the incidence of drug-related adverse events of Grade 3 or higher was 18.1%, limiting the clinical application of the combination 119. John *et al*. proposed that borussertib, an allosteric inhibitor of AKT, has a synergistic effect with MEK inhibitors in a preclinical model of the colorectum 120. Clarke *et al*. discovered that the MEK inhibitor cobimetinib and PI3K inhibitor pictilisib act synergistically in human CRC cells.

### Targeting the Wnt signaling pathway

Previous investigations identified the wingless integrated (Wnt) signaling pathway as a mechanism of resistance to selumetinib 121. Krishnamurthy *et al*. initiated a Phase-Ib clinical trial and found that a combination of selumetinib and cyclosporin A, an unconventional modulator of the Wnt signaling pathway, exhibited favorable tolerability and activity in CRC patients. The ORR in patients reached 76.9% 122. Jai-Hee *et al*. revealed that the β-catenin pathway inhibitor (NVP-TNS656) could overcome resistance induced by MEK inhibitors in *KRAS*-mutant CRC cells (Figure 2) 123.

### Targeting the JAK-STAT signaling pathway

Sandra *et al*. found that activation of Janus kinase 1/2 (JAK1/2)-dependent signal transducer and activator of transcription 3 (STAT3) can mediate the resistance to MEK inhibitors in *KRAS*-mutant CRC models. Notably, treatment with MEK inhibitors alone also leads to the activation of c-MET. Inhibition of either c-MET or JAK1/2, using compounds including AZD1480 and TG1038, shows the potential to enhance the sensitivity to MEK inhibitors (Figure 2).

## Reversal of KRAS inhibitor resistance by targeting upstream signaling pathways

Several studies indicate that *KRAS^G12C^*-mutant CRC retains the sensitivity to upstream RTK signaling. Upon the activation of RTK, GRB2 engages with the GEF SOS1 to form a complex that is associated with the cell membrane-bound KRAS protein, thus promoting the activation of KRAS. Consequently, more investigations have focused on the synergistic potential of combining KRAS inhibitors with agents targeting these upstream signaling pathways. Such combinatorial approach has shown promise in improving therapeutic efficacy in *KRAS*-mutant CRC.

### RTKs

RTKs constitute a distinct subclass of tyrosine kinases, which are instrumental in facilitating intercellular communication and regulating a diverse array of intricate biological processes. These include cellular growth, motility, differentiation, and metabolic activities. Upon activation, RTKs recruit and modulate various downstream signaling cascades, such as the RAS/MAPK, PI3K/AKT, and JAK2/STAT pathways 124. The activation of RTKs has been identified as a significant mechanism of resistance in patients with *KRAS*-mutant CRC. Clinical studies aimed at overcoming the resistance of KRAS inhibitors are also underway. Robin's team conducted clinical trials to evaluate the efficacy of MEK inhibitors in the downstream signaling pathway of KRAS in combination with pan-HER-2 inhibitors in *KRAS^G12C^*-mutant tumor groups. Still, the combination therapy was found to confer no apparent benefit in CRC, and the toxicity was severe 125. In addition, given the feedback activation of EGFR, several clinical researchers are exploring the efficiency of KRAS^G12C^ inhibitors combined with EGFR monoclonal antibodies. Multiple clinical studies implied that KRAS^G12C^ inhibitors combined with EGFR monoclonal antibodies show promising efficacy in patients with *KRAS*-mutant CRC, with manageable safety profiles 126-130. Table 2 summarizes the current five clinical studies on the combination of KRAS inhibitors with EGFR monoclonal antibodies in CRC patients. Future large-scale Phase-III clinical trials should further study the feasibility of combination treatment strategies. These results imply that targeting EGFR significantly improves the resistance of KRAS^G12C^ inhibitors in CRC patients.

### SHP2 inhibitors

SHP2 is a non-receptor protein tyrosine phosphatase that integrates growth and differentiation signals from RTKs into the RAS/MAPK cascade. SHP2 encoded by the protein tyrosine phosphatase non-receptor type 11 (PTPN11) gene functions downstream of RTKs to facilitate the physiological activation of KRAS 131. SHP2 also promotes nucleotide exchange in KRAS proteins 132. SHP2 activates RAS/MAPK and PI3K pathways by promoting KRAS-GTP. Inhibition of SHP2 reduces the circulation of KRAS^G12C^, which, together with SOS1, leads to a shift from an inactive GDP-bound pattern to an active GTP-bound pattern. Therefore, targeting SHP2 can overcome KRAS inhibitor resistance. Chen *et al*. proposed that SHP2 inhibitors block the feedback activation of downstream signaling pathways mediated by wild-type RAS, which exerts a synergistic effect with KRAS^G12C^ inhibitors and significantly suppresses the phosphorylation of downstream ERK 133. SHP2 inhibitor TNO155 has been reported to show synergistic effects with KRAS^G12C^ inhibitors 134. SHP2 inhibitors block RTK and MAPK-driven tumor cell proliferation 133 and inhibit the activation of RTK-driven feedback after treatment with KRAS inhibitors 135. The SHP2 inhibitor JC-010a eliminates RTK/SHP2-mediated resistance to selumetinib in *KRAS*-mutant cancers 136. Recently, Alexander's team identified a novel SHP2 inhibitor, GDC-1971, which exhibited significant antitumor ability in a mouse xenograft model of NSCLC and exerted a synergistic antitumor effect when combined with KRAS inhibitors 137. A Phase-I clinical study is now ongoing to assess the potency of divarasib combined with GDC-1971 in patients with *KRAS*-mutant solid tumors 132.

### SOS1 inhibitors

SOS1 as a type of GEF which is positioned upstream of KRAS plays a pivotal role in converting inactive KRAS proteins into their active GTP-bound state. Targeting SOS1 suppresses the activation of the RAS signaling pathway 83. Furthermore, SOS1 is essential for the negative feedback regulation of the KRAS pathway; thus, utilizing a combination of KRAS inhibitors and SOS1 inhibitors in *KRAS*-mutant CRC patients may significantly reverse drug resistance. BI-3406, a selective KRAS-SOS1 interaction inhibitor, significantly ameliorates MEK inhibitor resistance in *KRAS^G12C^*-mutant CRC cell lines and PDX models 138. In addition, the SOS1 inhibitor MRTX0902, when combined with the KRAS^G12C^ inhibitor adagrasib, has shown synergistic antitumor activity in PDX models of *KRAS^G12C^*-mutant pancreatic cancers. The clinical efficacy of this combination in* KRAS^G12C^*-mutant CRC patients remains to be confirmed 139. Furthermore, targeted degradation of SOS1 may yield superior efficacy compared to conventional inhibition. Currently, targeted degradation of SOS1 alone exhibits significant antitumor activity in *KRAS^G12C^*-mutant CRC and demonstrates synergistic anti-tumor activity with KRAS^G12C^ inhibitors 140, 141.

### Farnesyltransferase inhibitors

KRAS protein is located on the inner side of the cell membrane. This process requires farnesylation modification, which adds farnesyl groups to protein molecules under the action of farnesyltransferase. Hence, farnesyl transferase inhibitors may affect the activation of downstream signaling pathways mediated by KRAS protein. Marcell *et al*. identified that* KRAS*-mutated lung adenocarcinoma is sensitive to farnesyltransferase inhibitors. Alan *et al*. further confirmed that it has excellent clinical activity in HRAS-driven head and neck squamous cell carcinoma 142-144. Recently, a combination of additional KRAS^G1C^ inhibitors, sotorasib, and farnesyl-transferase inhibitors, tipifarnib, shows synergism in lung, colorectal, and pancreatic adenocarcinoma cellular models 145.

## Other strategies

### TEAD inhibitors

The Hippo pathway is a crucial growth control pathway conserved across species, negatively regulating the closely associated transcriptional coactivator paralogs YAP1 and TAZ. Overexpression or activation of YAP1 and TAZ can overcome the dependency of cancer cells on mutant *KRAS*, resulting in the proliferation of cancer cells 146, 147. Overexpression of YAP1-TAZ exerts proliferative effects on various tumor mutations, which may be related to the resistance to KRAS inhibitors. Several studies found that inhibition of YAP/TAZ or its transcription factor TEAD can overcome resistance to KRAS^G12C^ inhibitors. Edwards *et al*. developed TEAD inhibitors, including VT103, VT104, and VT107. All of which exert synergistic effects when used in conjunction with KRAS^G12C^ inhibitors 105, 148-150.

### Pan-RAS inhibitors

Dongsung *et al*. developed a pan-KRAS inhibitor, BI-2865, which can block nucleotide exchange to impede the activation of both wild-type *KRAS* and multiple *KRAS* mutants 151. Additionally, another pan-KRAS inhibitor, RMC-6236, has shown promising antitumor activity 152. Recently, it has been reported that a novel pan-RAS translation inhibitor 15a also suppresses the proliferation of *KRAS* mutation-driven tumor cells 153. Additionally, Mallika's team designed a new small-molecule compound, RMC-7977, with broad inhibition of the active pattern of wild-type and mutants *KRAS*, *HRAS*, and *NRAS*. RMC-7977 exhibited optimal efficacy against *RAS*-addicted neoplasms characterized by diverse *RAS* genotypic profiles in preclinical oncology models, showing pronounced activity in models harboring *KRAS* codon 12 mutations (*KRAS^G12X^*) 154. Subsequently, Olive *et al*. confirmed the significant antitumor efficacy of RMC-7977 in preclinical models of pancreatic cancer and revealed that overexpression of YAP promotes resistance to RMC-7977 in pancreatic cancer 155.

### Targeting metabolism-related pathways

Studies discovered that *KRAS*-mutant cells improve the expression of proteins that participate in glycolysis, the non-oxidative branch of the pentose phosphate pathway, glutamine metabolism, and the phosphoserine-biosynthesis pathway 156, 157.

#### Targeting glucose metabolism

*KRAS*-mutant tumors are more reliant on the glycolytic pathway for energy production, driving the conversion of glucose to pyruvate and ultimately to lactate through upregulation of key enzymes in glycolysis 158, 159. Targeting glycolysis has emerged as a promising strategy for *KRAS*-driven tumors. Li's research team found that BNBZ induces metabolic reprogramming in *KRAS*-mutant CRC cells by inhibiting glycolytic flux, thereby suppressing the tumor cell growth. The underlying mechanism primarily involves the inhibition of the key glycolytic enzyme HK2, which leads to impaired cell proliferation and increased production of reactive oxygen species (ROS). Additionally, BNBZ promotes apoptosis by upregulating Bax and downregulating Bcl-2^160^. Johanna found that simultaneous inhibition of the glucose transporter GLUT1 and the ATR/CHK1 cell-cycle checkpoint signaling axis induces S-phase specific genotoxic stress and apoptosis in various *KRAS*-mutant cell lines, including CRC, both *in vitro* and *in vivo* 161.

#### Targeting glutamine metabolism

Glutamine is critical to the survival and progression of *KRAS*-mutant CRC. Solute carrier 25 member 21 (SLC25A21) is an oxo-dicarboxylate carrier responsible for transporting 2-oxo adipate (2-OA) and α-ketoglutarate (α-KG) across the inner mitochondrial membrane 162. The survival and progression of KRAS-mutant CRC depend on glutamine (Gln). Downregulation of SLC25A21 inhibits the mitochondrial export of Gln-derived α-ketoglutarate (α-KG), thereby enhancing glutamine metabolism. This response, characterized by the cessation of α-KG export due to the downregulation of SLC25A21, promotes downstream oxidative decarboxylation reactions and GTP production, leading to sustained KRAS activation in KRAS-mutant CRC, which drives cell proliferation. Increasing the SLC25A21 expression can induce cell death in KRAS-mutant CRC 163. Another study indicated that SLC7A5 maintains intracellular amino acid levels through transcriptional and metabolic reprogramming following KRAS activation, further supporting the demand of the tumor cells for extensive protein synthesis. Additionally, the loss of SLC7A5 can inhibit the mTOR signaling. Targeting the glutamine transporter SLC7A5 suppresses the proliferation of *KRAS*-mutant cancers. When combined with mTOR inhibitors, this approach shows a synergistic effect 164. These findings indicate that targeting metabolic pathways with KRAS inhibitors may reverse drug resistance.

### Epigenetic-based treatment strategies

For a long time, epigenetic and genetic alterations have been thought of as two independent mechanisms participating in tumorigenesis 165. Epigenetics assumes a crucial role in tumor progression 166. Among them, inhibition of histone deacetylases (HDACs) has been found to promote the effects of RAS pathway inhibitors in numerous tumor types 167, 168. HDAC-mediated histone deacetylation is a post-translational modification process involving the lysine residues of histones in the nucleosome, which affects the chromatin structure and consequently regulates the gene expression. In malignant tumors, the activity of HDAC is often upregulated to further suppress the expression of tumor suppressor genes, which positions HDACs as attractive therapeutic targets 168. Selumetinib and the HDAC inhibitor vorinostat show synergistic antitumor activity in *KRAS*-mutant CRC cell lines 169. Hendrik *et al*. found that *KRAS*-mutant CRC cells induce the expression of hypoxia-inducible factor-1α and 2α. The microtubule agent dolastatin 10 and the Class I HDAC inhibitor largazole suppress the oncogenic *KRAS* and HIF pathways, inducing tumor regression 170. Additionally, the HDAC6 inhibitor C1A was found to modulate autophagy substrates in *KRAS*-positive CRCs, inducing cell death 171. These findings imply that co-targeting HDACs and KRAS could be a valuable avenue for future research and therapeutic strategies. In addition, studies reported that inhibitors of the histone methyltransferase EZH2 exert a synergistic antitumor effect when combined with RAS pathway inhibitors in *KRAS*-mutant CRC. The combined use of both can inhibit the WNT pathway, leading CRC to reach a more differentiated cellular state, thereby making it more sensitive to inhibitors 172.

## The combination of KRAS inhibitors and immune checkpoint inhibitors

**Figure 4:**
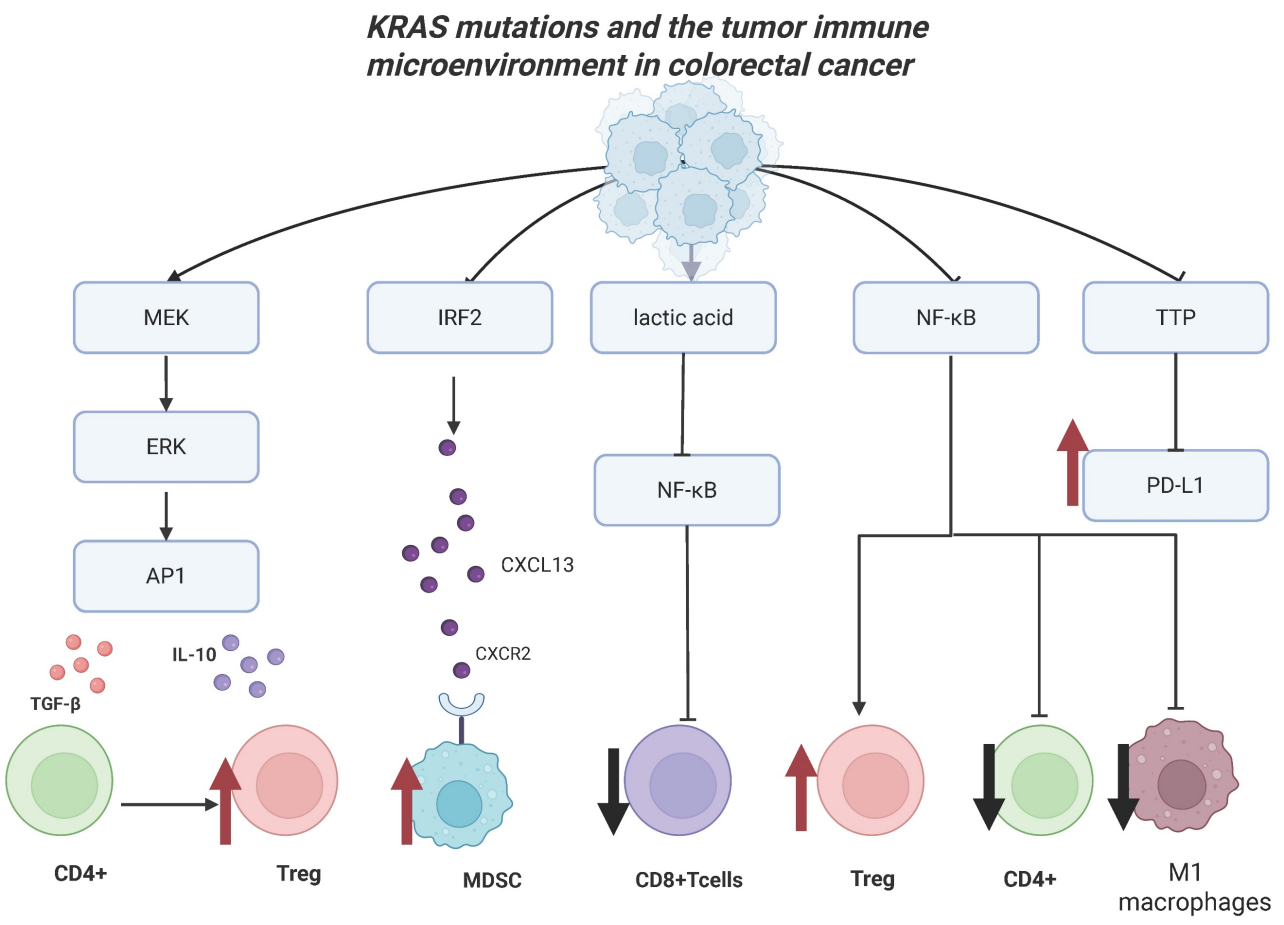
*KRAS* mutations and the tumor immune microenvironment. Effect of *KRAS* mutations on tumor microenvironment in colorectal cancer. *KRAS*-mutated tumor cells can convert CD4+ T cells into regulatory T cells through the MEK-ERK-AP1 signaling pathway; recruiting MDSCs to the tumor microenvironment by upregulating the expression of IRF2; inhibiting NF-κB signaling by upregulating the expression of lactic acid, further reducing the number of CD+ T cells; inhibiting the NF-κB signaling pathway, increasing the number of regulatory T cells, reducing the number of CD4^+^ T cells and the number of M1 macrophages, Inhibiting the expression of TTP and upregulates the expression of PD-L1. Created in BioRender. Li, J. (2025) https://BioRender.com/vzgd3pj.

The emergence of immunotherapy has remarkably converted the therapeutic landscape of CRC. Considerable attention has been paid to the collaborative application of immune checkpoint inhibitors (ICIs) in conjunction with drugs that disrupt the activation of oncogenic signaling cascades. Previous studies reported that *KRAS*-mutant CRC patients are associated with an immunosuppressive tumor microenvironment (TME) (Figure 4).

### Regulation of immune cell subsets

*KRAS*-mutant CRC sensitizes tumor-specific cytotoxic CD8+ T-cells to activation-induced cell death via NF-κB inactivation (Figure 4) 173. Liu *et al*. also discovered that NF-κB and T-cell receptor signaling cascades were significantly suppressed in patients with *KRAS* mutations. Additionally, Tregs were increased while activated CD4 memory T cell and macrophage M1 could be reduced in* KRAS*-mutant CRC (Figure 4) 174.

### Effect on immune checkpoint molecules

Oncogenic RAS signaling upregulates the expression of PD-L1 mRNA in CRC. This process is regulated by the AU-rich element-binding protein tritetrapeptide TTP, which negatively regulates the expression of PD-L1 mRNA. MEK signaling downstream of oncogenic RAS suppresses the expression of TTP, upregulating the PD-L expression to evade immune surveillance (Figure 4) 175. Additionally, another study found that *KRAS*-mutant patients have higher expression of CTLA-4 mRNA and poorer prognosis 176.

### Cytokine and chemokine production

The mutant KRAS protein can drive the expression of diverse cytokines and chemokines, thus facilitating the formation of an immune-heterogeneous TME 177. Specifically, the activation of the MEK-ERK-AP1 pathway by mutated *KRAS* induces tumor cells to secrete interleukin-10 (IL-10) and transforming growth factor-β1 (TGF-β1). This expedites the transformation of CD4+ T cells into regulatory T cells (Tregs), which inhibit the tumor immunity (Figure 4). Moreover, the oncogenic *KRAS^G12D^* variant has been found to inhibit the expression of interferon regulatory factor 2 (IRF2), which negatively regulates the CXCL3 expression. *KRAS^G12D^*-mediated inhibition of IRF2 ultimately causes the high expression of CXCL3, which can bind to CXCR2 on myeloid-derived suppressor cells (MDSCs) and promotes its migration to the TME 178 (Figure 4). Moreover, the TGF-β signaling pathway functions as a mediator of immune escape and tumor invasion in *KRAS*-mutant CRC 179, 180.

### Measures for combination therapy

Given the immunosuppressive nature of *KRAS*-mutant tumors, combining ICIs with KRAS-targeted therapies holds promise. KRAS inhibitors can also remodel the TME, leading to the formation of pro-inflammatory TME, and resulting in the remodeling of the tumor immune microenvironment in *KRAS*-mutant cancers 181.

Preclinical studies show that the combination of KRAS inhibitors and ICIs significantly improves survival in CRC models. In the *KRAS^G12C^*-mutant CT26 syngeneic mouse model, adagrasib reduces intra-tumoral MDSCs and enhances the infiltration of M1-polarized macrophages, CD4+ T cells, dendritic cells, and CD8+ T cells. The combination of ICI and KRAS^G12C^ inhibitor significantly increases the survival ratio of mice in the subcutaneous model of CT-26 syngeneic CRC 182. In a *KRAS^G12D^*-mutant CRC mouse model, treatment with RMC-9805 increases T cell infiltration, reduces the infiltration of M2-like macrophages and MDSCs, significantly decreases the expression of immune checkpoint molecules, and markedly increases the MHC-I expression. RMC-9805 exerts a synergistic antitumor effect with ICIs, significantly inhibiting tumor proliferation 183.

Clinical studies confirmed that KRAS^G12C^ inhibitors in conjunction with ICIs elicit substantial antitumor activity in NSCLC patients, achieving an ORR of 63% in individuals with a programmed cell death 1 ligand 1 (PD-L1) tumor proportion score (TPS) of greater than, or equal to, 50%. The median duration of response does not reach (95% CI, 12.6 - NE) 184. The efficiency of KRAS^G12C^ inhibitors combined with ICIs in* KRAS*-mutant CRC patients has not, at time of writing, been studied. Combining ICIS and KRAS inhibitors warrants further exploration 185.

Hapten-based immunotherapy has the potential to overcome drug resistance 186-188. Zhang *et al*. found that the covalent modification of KRAS (G12C) on tumor-specific cysteine residues causes the formation of haptenized peptides that can be presented by MHC-I molecules. These peptides were identified by the recombinant antibody P1A4, which selectively activates a cytolytic T-cell response to target and eliminate tumor cells. This approach provides a novel strategy for reversing drug resistance in cancer therapies 189.

Furthermore, post-translational modifications of proteins significantly affect the TME. Kim *et al*. found that statins enhance the cross-priming capabilities of dendritic cells, thus promoting immunogenic cell death of CD8+ T cells targeting *KRAS*-mutant tumors. This indicates that statins have synergistic antitumor effects when used in combination with ICIs 190.

The interaction between mutated *KRAS* and the immune microenvironment is a key determinant of tumorigenesis, progression, and treatment response. Targeting this pathway can not only directly suppress the proliferation of tumor cells but also enhance antitumor immune responses by remodeling the immune microenvironment. The combination of KRAS inhibitors with ICIs holds promise as a new treatment option for patients with *KRAS*-mutant CRC.

## Discussion

Significant progress has been achieved in the research of KRAS inhibitors. In addition to the two approved KRAS^G12C^ inhibitors (sotorasib and adagrasib), non-covalent selective inhibitors targeting KRAS^G12D^ (MRTX1133 and HRS-4642), the inhibitor targeting KRAS^G12V^ (EFTX-G12V), and pan-RAS inhibitors (BI-2865, RMC-6236, and RMC-7977) are currently at different stages of clinical development. Resistance remains a major concern, as a single inhibitor is inadequate for effective tumor eradication, underscoring the need for novel therapeutics and combination strategies to overcome resistance 191. Current combination treatment strategies primarily focus on KRAS^G12C^ inhibitors. The combination of KRAS^G12C^ inhibitors with EGFR monoclonal antibodies may yet become a standard treatment for CRC patients. However, the efficacy of combinations involving SOS1, SHP2, and farnesyltransferase inhibitors has not yet been validated in clinical studies. A preclinical study confirmed that in CRC models resistant to adagrasib, combining adagrasib with SOS1 or SHP2 inhibitors shows comparable efficacy, both surpassing the effectiveness of adagrasib combined with EGFR monoclonal antibodies 192. Although both therapeutic approaches demonstrate similar efficacy, SOS1 inhibition may mitigate some of the pleiotropic effects associated with SHP2 inhibitors, potentially improving clinical tolerability. Studies in mouse models have validated that KRAS^G12D^ inhibitors, KRAS^G12V^ inhibitors, and the Pan-RAS inhibitor exhibit synergistic anti-tumor effects when combined with EGFR monoclonal antibodies.

The combination therapy of KRAS inhibitors with ICIs is also being actively investigated 48, 182. Several ongoing clinical studies are exploring the conjunction of ICIs with KRAS^G12C^ inhibitors (Table 3). Recent research utilizing spatial transcriptomics has revealed that the TME in *KRAS*-mutant CRC patients is suppressed, with specific immune gene signatures linked to poorer OS. The study identified an upregulation of CD40, CTLA4, ARG1, STAT3, IDO, and CD274 in the TME of these patients, which may serve as characteristics of immune suppression 193. Therefore, the clinical effect of combined treatment is expected to be promising.

Patient selection is critical for analyzing the efficacy of KRAS inhibitors in CRC. Molecular profiling of CRC patients supports precision therapy, as factors like *NRAS*, *BRAF*, and *PIK3CA* mutations can affect treatment responses. Therefore, a comprehensive assessment of multiple biomarkers is essential for developing treatment plans. Next-generation sequencing (NGS) technology provides comprehensive information on the tumor genome, helping to identify mutations associated with resistance 194.

Although many studies have found that targeting metabolism-related pathways may alleviate resistance, treating cancer metabolism as a therapeutic target still faces significant challenges. One major challenge is how to eliminate tumor cells while maintaining cellular metabolic homeostasis. Additionally, tumor heterogeneity and the differences in metabolic profiles among patients highlight the importance of precision medicine. Development of metabolic biomarkers could aid in predicting patient responses to these therapies, thus facilitating more personalized, effective treatment options. Protein homeostasis also plays a crucial role in tumor resistance. Clinical trials discovered that the IRE1α inhibitor ORIN1001 exhibits antitumor activity in various solid tumors, including CRC, highlighting the potential to target protein homeostasis.

In CRC cells, cellular plasticity is often regulated by epigenetic mechanisms, implying that targeting key epigenetic regulators, in addition to traditional oncogenic signaling pathways, may represent a potential therapeutic strategy. Current research has identified that HDAC and EZH2 inhibitors exert synergistic antitumor effects when combined with RAS pathway inhibitors. Further exploration of this area of study is warranted.

Despite certain advances in the research into *KRAS*-mutant CRC, current studies still face several limitations: first, many preclinical studies are based on cell lines or cell-derived xenograft (CDX) models. These models may exhibit significant differences in biological characteristics and drug responses compared to human tumors, thereby limiting the generalizability of their results. Future preclinical research should focus on patient-derived xenograft (PDX) models, and integrate new technologies like organoids, organ-on-a-chip systems, and artificial intelligence to achieve high-throughput precision drug screening 195. This approach aims to deeply elucidate resistance mechanisms and ultimately match each patient with the most effective personalized treatment. Second, the safety of KRAS inhibitor combination therapies is a major challenge. Current regimens mainly pair them with EGFR monoclonal antibodies; existing Phase I-II studies show manageable safety, but long-term profiles need large-scale trials. Combining with other targeted/immunotherapies is constrained by higher toxicity. A 1,029-patient network meta-analysis found grade 3+ AEs occurred in 26% of patients (monotherapy) vs 35% (combination therapy). Notably, KRAS^G12C^ inhibitor combined with ICIs raised this to 50% (with liver toxicity a concern 196), while Codebreak 100/101 showed sotorasib + anti-PD-(L)1 boosted grade 3-4 TRAEs, mainly elevated transaminases. Future research should focus on exploring low-toxicity combination strategies and identifying predictive biomarkers of adverse events. Finally, the clinical translation process faces numerous challenges. Factors such as patient heterogeneity and the complexity of the TME can significantly impact treatment efficacy. Therefore, determining how to accurately select suitable patients to maximize therapeutic benefits is a crucial direction for future research.

KRAS inhibitors have demonstrated promise in CRC treatment, however, they remain constrained by challenges including patient selection, mechanisms of resistance and TRAEs. The integration of the application of biomarkers with emerging technologies such as next-generation sequencing and spatial transcriptomics holds potentials to enhance the efficacy of KRAS inhibitors; meanwhile, optimizing strategies to reduce adverse reactions can further mitigate treatment risks. Together, these efforts will bring safer, and more effective, new treatment options for CRC patients.

## Figures and Tables

**Table 1:** Ongoing or planned registered clinical trials of KRAS inhibitors in advanced solid tumors or colorectal cancer listed on clinicaltrials.gov (query date: September 2025)

Drug	Phase	Cancer type	ClinicalTrials.gov Identifier	State
RMC-6291	Phase1	Advanced Solid Tumors	NCT05462717	Recruiting
GFH925	Phase1/2	Advanced Solid Tumors	NCT05005234	Recruiting
ZG19018	Phase1/2	Advanced Solid Tumors	NCT06237400	Recruiting
BEBT-607	Phase 1	Advanced Solid Tumors	NCT06117371	Recruiting
D3S-001	Phase 1	Advanced Solid Tumors	NCT05410145	Recruiting
HBI-2438	Phase 1	Advanced Solid Tumors	NCT05485974	Recruiting
FMC-376	Phase1/2	Advanced Solid Tumors	NCT06244771	Recruiting
JAB-21822	Phase1/2	Advanced Solid Tumors	NCT05009329	Recruiting
MRTX849	Phase 1	Advanced Solid Tumors	NCT05263986	Active, not recruiting
SY-5933	Phase 1	Advanced Solid Tumors	NCT06006793	Recruiting
GEC255	Phase 1	Advanced Solid Tumors	NCT05768321	Recruiting
LY3537982	Phase 1	Advanced Solid Tumors	NCT06235983	Not yet recruiting
HS-10370	Phase1/2	Advanced Solid Tumor	NCT05367778	Recruiting
BPI-421286	Phase 1	Advanced Solid Tumors	NCT05315180	Unknown
HRS-4626	Phase 1	Advanced Solid Tumors	NCT05533463	Recruiting
MRTX-1133	Phase1/2	Advanced Solid Tumors	NCT05737706	Recruiting
ASP4396	Phase1	Advanced Solid Tumors	NCT06364696	Not yet recruiting
RMC-9805	Phase1	Advanced Solid Tumors	NCT06040541	Recruiting

Table 1 summarizes ongoing or planned clinical trials exploring the efficacy of KRAS inhibitor monotherapy in solid tumors. Data from ClinicalTrials.gov.

**Table 2:** Clinical trials evaluating the efficacy of KRAS inhibitor combined with ICIs in CRC patients.

Study	Phase	Intervention	ORR	mPFS (month)	3-4TRAE
NCT04449874 126	Ib	Divarasib +Cetuximab	62.5%	8.1	44.8%
NCT04185883 127	Ib	Sotorasib+Panitumumab	30.0%	5.7	27%
NCT03785249 128	I-II	Adagrasib+ Cetuximab vs. Adagrasib	46% vs.19%	6.9 vs. 5.6	16% vs. 34%
NCT05198934 129	III	Sotorasib(960mg/240mg) + Panitumumab vs. standard-care (trifluridine-tipiracil or regorafenib)	26.4% vs. 5.7% vs.0%	5.6 vs. 3.9 vs.2.0	35.8% vs. 30.2% vs. 43.1%
NCT04585035 130	II	Garsorasib + Cetuximab vs. Garsorasib	45.2% vs. 19.2%	7.5 vs. 5.5	19.2% vs.14.3%

Table 2 summarizes the efficacy and safety of KRAS inhibitors in combination with EGFR monoclonal antibodies. Data from ClinicalTrials.gov.

**Table 3:** Ongoing or planned registered clinical trials of KRAS inhibitors, in combination with other medications in advanced solid tumors or colorectal cancer listed on clinicaltrials.gov (query date: September 2025)

Drug	Target	Combination	Phase	Cancer type	ClinicalTrials.gov Identifier	State
ASP3082	EGFR	Cetuximab	Phase 1	Advanced Solid Tumors	NCT05382559	Recruiting
MRTX849	PD-1/EGFR	Pembrolizumab, Cetuximab, Afatinib	Phase 1/2	Advanced Solid Tumors	NCT03785249	Recruiting
JDQ443	MEK/CDK4/6 EGFR	Trametinib, Ribociclib, cetuximab	Phase Ib/II	Advanced Solid Tumors	NCT05358249	Recruiting
Adagrasib	SHP2/EGFR	BMS-986466 with or without Cetuximab	Phase1/2	Advanced Solid Tumors	NCT06024174	Active, not recruiting
Sotorasib	EGFR	Panitumumab	Phase2	Advanced Solid Tumors	NCT05993455	Active, not recruiting
Sotorasib	PD1/L1	Anti PD-1/L1, Midazolam	Phase 1/2	Advanced Solid Tumors	NCT03600883	Active, not recruiting
Adagrasib	CDK4/6	Palbociclib	Phase1	Advanced Solid Tumor	NCT05178888	Active, not recruiting
QTX3034	EGFR	Cetuximab	Phase1	Advanced Solid Tumors	NCT06227377	Recruiting
BI- 1823911	SOS1	BI-1701963 Midazolam	Phase1	Advanced Solid Tumors	NCT04973163	Active, not recruiting
Drug	Target	Combination	Phase	Cancer type	ClinicalTrials.gov Identifier	State
IBI315	EGFR	Cetuximab	Phase 1	KRAS G12C Mutated Metastatic CRC	NCT05497336	Unknown status
Sotorasib	MEK SHP2 EGFR PD-1 EGFR / / / / PD-L1 CDK4/6 / / SHP2 / / SOS1 PD-1 mTOR	Trametinib, RMC-4630, Afatinib, Pembrolizumab Panitumumab, Carboplatin, pemetrexed, docetaxel, paclitaxel, Atezolizumab, Palbociclib, MVASI® (bevacizumab-awwb), TNO155, FOLFIRI, FOLFOX, BI 1701963, AMG 404, Enerolimus	Phase 1	Advanced Solid Tumors	NCT04185883	Active, not recruiting

Table 3 summarizes ongoing or planned clinical trials exploring the efficacy of KRAS^G12C^ inhibitors combined with other treatments in solid tumors. Data from ClinicalTrials.gov.

## Abbreviations

AP1: activating protein-1
CDK: Cyclin-dependent kinase
CDX: cell-derived xenografts
CsA: cyclosporin A
CYPA: cyclophilin A
CRC: colorectal cancer
EGFR: epidermal growth factor receptor
ERK: Extracellular regulated protein kinases
FDA: Food and Drug Administration
FGFR: Fibroblast Growth Factor Receptor
GAPs: GTPase activates proteins
GDP: Guanosine diphosphate
GEFs: Guaninenucleotide exchange factors
GRB2: growth factor receptor binding protein 2
GTP: Guanosine triphosphate
HVRs: highly variable regions
IGF-IR: Insulin-like Growth Factor 1 Receptor
IL-10: interleukin-10
JAK1/2: Janus kinase1/2
KEAP1: Kelch-like ECH-associated protein 1
KRAS: Kirsten rat sarcoma virus
MAPK: mitogen-activated protein kinase
MEK: mitogen-activated protein kinase kinase
mTOR: mechanistic target of rapamycin kinase
MYC: myc avian myelocytomatosis
NCSCL: non-small cell lung cancer
ORR: objective response rate
OS: overall survival
PAK: P21-activated kinase
PD-L1: programmed cell death 1 ligand 1
PDX: patient-derived xenografts
PDAC: Pancreatic ductal adenocarcinoma
PFS: progression-free survival
PI3K: phosphoinositide-3-kinase
PLK1: Polo-like Kinase 1
PROTAC: Proteolysis-Targeting Chimeras
PTPN11: protein tyrosine phosphatase non-receptor type 11
RB: Retinoblastoma gene
RFS: Recurrence-free survival
RTK: receptor tyrosine kinase
SHP2: Src homology-2 domain-containing protein tyrosine phosphatase-2
SOS1: son of sevenless homolog 1
STAT3: signal transducer and activator of transcription 3
TGF-β1: transform growth factor-β1
TPS: Tumor Proportion Score
TME: tumor microenvironment
Wnt: wingless integrated
